# Dual-Function Hydrogel Coating on Silicone Urinary Catheters with Durable Antibacterial Property and Lubricity

**DOI:** 10.3390/gels11020128

**Published:** 2025-02-10

**Authors:** Shuai Gao, Wei Zeng, Zheng Liu, Fanjun Zhang, Yunfeng Zhang, Xi Liu, Dimeng Wu, Yunbing Wang

**Affiliations:** 1 National Engineering Research Center for Biomaterials, College of Biomedical Engineering, Sichuan University, Chengdu 610065, China; shuai.gao@daxanmed.com (S.G.); zhfanjun@163.com (F.Z.); 17311480487@163.com (X.L.); 2 Chengdu Daxan Innovative Medical Tech. Co., Ltd., Chengdu 611137, China; eden_zengw@163.com (W.Z.); lz6988@163.com (Z.L.); zyf19900320@163.com (Y.Z.)

**Keywords:** silicone urinary catheter, hydrogel coating, antibacterial property, lubricity

## Abstract

Silicone urinary catheters are broadly employed in medical practice. However, they are susceptible to inducing catheter-associated urinary tract infections (CAUTIs) due to bacterial adherence to the catheter’s surface, and they exhibit a high friction coefficient, which can greatly affect their effectiveness and functionality. Thus, the development of a silicone urinary catheter with antibacterial properties and lubricity is in strong demand. We hereby developed a poly(vinyl acetate) carrier coating to load chlorhexidine acetate and applied a hydrogel coating primarily composed of polyvinylpyrrolidone (PVP) and poly(ethylene glycol) diacrylate (PEGDA), which was then coated onto the silicone urinary catheters and cured through a thermal curing process and could provide lubricity. Subsequently, we analyzed its surface characteristics and assessed the antibacterial property, lubricity, cytotoxicity, and potential for vaginal irritation. The findings from the Fourier transform infrared spectrometer (FTIR), scanning electron microscope (SEM), water contact angle (WCA), inhibition zone measurements, and friction coefficient analysis confirmed the successful modification of the silicone urinary catheter. Additionally, the outcomes from the cytotoxicity and vaginal irritation assessments demonstrated that the dual-function hydrogel coating-coated silicone urinary catheters exhibit outstanding biocompatibility. This study illustrates that the prepared silicone urinary catheters possess durable antibacterial properties and lubricity, which thus gives them broad clinical application prospects.

## 1. Introduction

Urinary catheters are among the most frequently utilized medical devices in hospitals [1,2,3,4]. Nonetheless, catheter-associated urinary tract infections (CAUTIs) are recognized as the most common category of hospital-acquired infections (HAIs), with about 80% of CAUTIs resulting from indwelling urinary catheters, which are ascribed to the adhesion and proliferation of bacteria on the surfaces of urinary catheters, and the follow-up formation of highly resistant biofilms [5,6,7,8,9]. The CAUTIs can result in increased healthcare expenses, prolonged hospital stays, and potentially greater morbidity and mortality rates [10,11,12,13]. Silicone rubber is widely utilized in medical applications to produce many medical devices because of its excellent biocompatibility, flexibility, and physiological stability, which make silicone rubber an ideal material for indwelling urinary catheters [14,15,16,17]. Despite these advantages, silicone rubber can cause extreme tissue trauma, postoperative pain, occlusion, and infection owing to the high dynamic friction coefficient. Consequently, it is crucial to guarantee that the silicone urinary catheter’s outer surface is adequately lubricated so as to reduce friction with the urethral tissues [18,19,20]. Additionally, the highly hydrophobic surface of silicone rubber can readily attract bacteria and other biological cells, leading to issues such as wound infections, inflammation, and other complications [21,22]. Therefore, there is a pressing need to establish effective surface functionalization methods to enhance the antibacterial properties and lubrication of silicone urinary catheters.

After decades of research, two antibacterial mechanisms, contact-killing and release-killing, have been formed [23,24,25,26]. Researchers have done a lot of work based on these two mechanisms to construct antibacterial coatings on silicone urinary catheters. Antonio Francesko et al. utilized layer-by-layer assembly of hyaluronic acid and cationic aminocellulose nanospheres to endow silicone urinary catheters with good antibacterial properties [27]. Vanessa C. Thompson et al. utilized the electron transfer (ARGET)–atom transfer radical polymerization (ATRP) method to construct a pDA-g-pMTAC coating on silicone urinary catheters, resulting in a obvious decrease in bacterial adhesion [28]. Matej Bračič and his co-workers modified the silicone tubes by incorporating colloidal polysaccharide complexes, which display slow-release leaching, antibacterial characteristics, and outstanding resistance to mechanical abrasion [29]. Rui Ding et al. used tannic acid and copper to develop metal–phenolic network coatings that provide antibacterial surfaces for silicone catheters; the modified catheters exhibited desirable biocompatibility and remarkable antibacterial properties [30]. Although previous researchers have made great progress in modifying silicone urinary catheters, there are still some issues, such as the complex processing process, the failure to combine lubricity and antibacterial properties well, and insufficient antibacterial properties. Therefore, it remains a challenge to efficiently and effectively impart the desired antibacterial properties and lubricity to silicone urinary catheters.

Chlorhexidine acetate is an antiseptic and preservative known for its wide-ranging antibacterial effectiveness against both Gram-negative and Gram-positive bacteria. It is utilized in various infection control procedures, such as systemic irrigation, oral hygiene, and surgical hand disinfection in critical care environments [31,32]. The antibacterial mechanism of chlorhexidine acetate is its ability to bind to the negatively charged bacterial membrane, leading to the disruption of the outer leaflet. Nonetheless, high concentrations of chlorhexidine acetate may cause irritation to the mucous membranes and induce cytotoxicity [33]. Polyvinyl pyrrolidone (PVP) exhibits excellent physiological inertness and biocompatibility, making it a commonly employed lubricating agent in hydrophilic lubrication coatings [34]. The hydrophilic properties of PVP hydrogel coatings facilitate catheter insertion by greatly minimizing friction between the catheter and ureteral tissue. This aspect is vital for the successful implantation of catheters in the human body and may contribute to improved patient comfort [35]. However, PVP demonstrates inadequate adhesion capabilities with the substrates, which restrict its long-term applications. Therefore, the preparation of PVP hydrogel coatings with sustained release of chlorhexidine acetate and application of the coating on silicone urinary catheters may address the aforementioned issues.

**Scheme 1:**
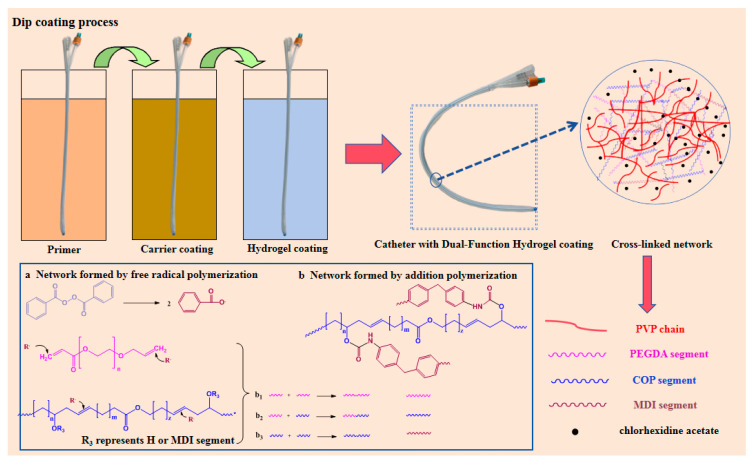
Schematic illustration of experimental design, process and mechanisms.

In this article, we provide an efficient and effective surface modification method to prepare a dual-function hydrogel coating with release-killing and lubricity on silicone urinary catheters. The dual-function hydrogel coatings mainly contain a poly(vinyl acetate) carrier coating, which controls the release of chlorhexidine acetate, and the hydrogel coating, which is described in our previous report [36]. Under thermal curing conditions, cross-linked networks could be established to securely incorporate the PVP (Scheme 1). The surface of the silicone urinary catheters or sheets was analysed by FTIR, SEM and WCA tests. The inhibition zone and friction coefficient were measured to evaluate the antibacterial properties and lubricity of the silicone urinary catheters, respectively. The MTT assay was subsequently employed to evaluate the silicone urinary catheters’ cytotoxicity. Finally, modified silicone urinary catheters were produced using industrial equipment, and an assessment of vaginal irritation was conducted. This work provides a efficient and effective method for scalable production of silicone urinary catheters with antibacterial properties and lubricity.

## 2. Results and Discussion

We modified silicone urinary catheters and sheets using a three-step dipping procedure, followed by a thermal curing process. The cured primer layer introduced high-polar carbonyl and hydroxyl functional groups to the surface of the silicone, which could increase the surface energy and wettability of the silicone catheters or sheets and then increase the hydrogel coating’s adhesion to the substrates. The following poly(vinyl acetate) carrier coating loaded chlorhexidine acetate and further improved the silicone’s surface energy. Thus, the dual-function hydrogel coating could be uniformly applied to the surface of silicone catheters or sheets. The formed network was capable of incorporating PVP into the coating and exhibited strong adhesion to the silicone urinary catheter. In the Results and Discussion section, silicone urinary catheter or sheets without modification were named as pristine, while the modified silicone urinary catheters or sheets were named as DF-0.5 (0.05%wt) or DF-1 (0.1%wt) according to the corresponding chlorhexidine acetate amount in the poly(vinyl acetate) carrier coating.

### 2.1. Surface Characterization

#### 2.1.1. FTIR Test

**Figure 1:**
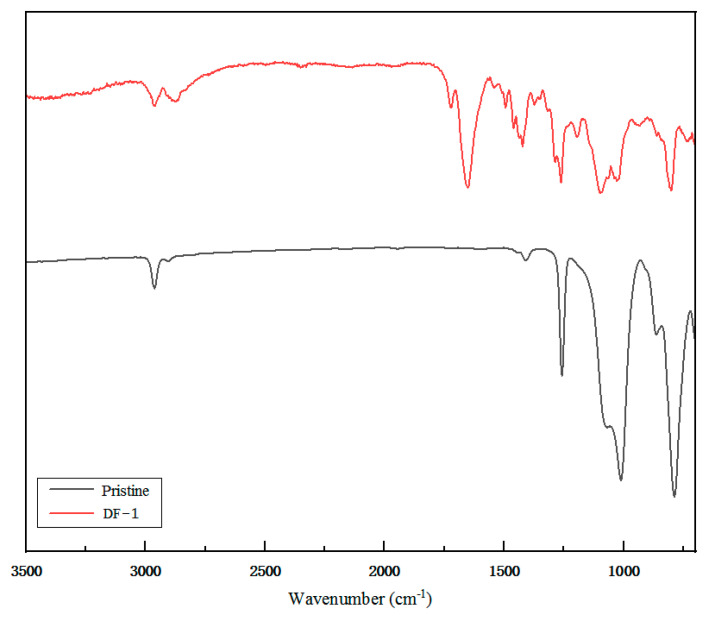
The FTIR images of pristine and modified silicone sheets.

The FTIR test was used to analyse the surface functional group changes of the dual-function hydrogel coating-modified silicone sheets. The FTIR images of the silicone sheets are illustrated in Figure 1. It was observed that the typical absorption peaks of the pristine silicone sheets encompassed the Si-C stretching vibration peak at 787 cm^−1^, the Si-O stretching vibration peak at 1076 cm^−1^, the Si-CH_3_ absorption peak at 1256 cm^−1^, and the C-H asymmetric stretching vibration peak at 2963 cm^−1^. Following the application of a dual-function hydrogel coating, the initial typical absorption peaks of the pristine silicone sheets were significantly diminished. The peaks observed in the modified silicone sheets were outlined as follows: 1723 cm^−1^, corresponding to the stretching vibration of carbonyl of cured MDI prepolymer; 1652 cm^−1^, for the stretching vibration peak of the PVP’s -CON- group; 1420 cm^−1^, for the N-H stretching vibration peak; 1288 cm^−1^, for the C-N stretching vibration peak; and 2960 cm^−1^ and 2871 cm^−1^, representing the C-H asymmetric stretching vibration and symmetric stretching vibration peak, respectively. Through an infrared spectra test, it could be concluded that after modification with dual-function hydrogel coating, a polyurethane film that includes PVP and PEGDA segments was created on the silicone sheet surfaces, signifying effective surface modification.

#### 2.1.2. SEM Test 

**Figure 2:**
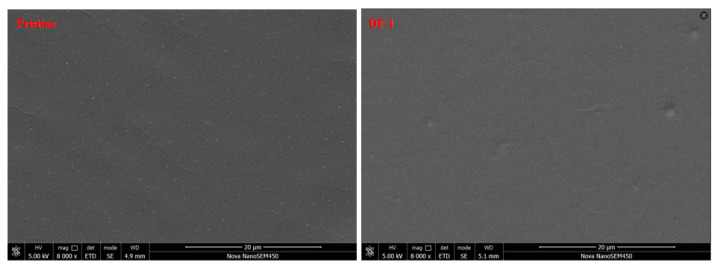
The SEM images of silicone urinary catheters.

Using SEM, we examined the surface morphology of the silicone urinary catheter modified with a dual-function hydrogel coating, with the pristine silicone urinary catheter serving as the control (Figure 2). The pristine silicone urinary catheter exhibited a relatively rough surface, whereas the surface of the silicone urinary catheter modified with the dual-function hydrogel coating was smooth, which was ascribed to the bonded hydrogel coating. Meanwhile, the improved surface smoothness may reduce bacterial adhesion [37].

#### 2.1.3. WCA Measurement

**Figure 3:**
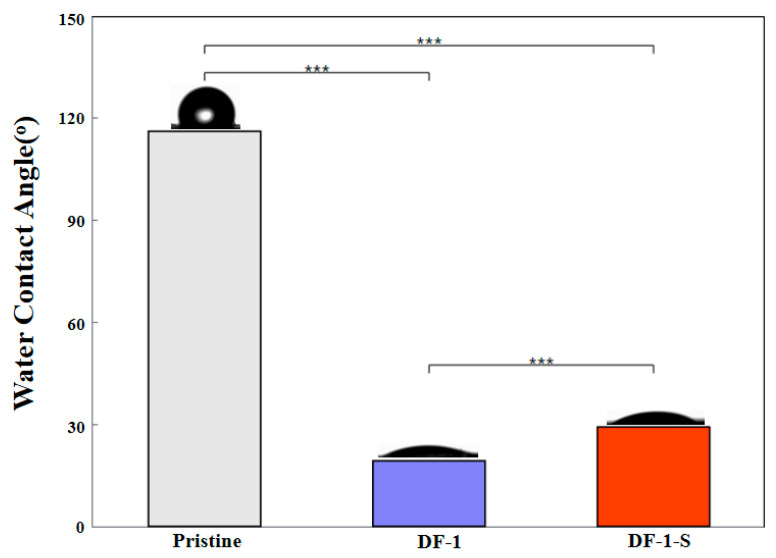
WCA of pristine and dual-hydrogel coating-modified silicone rubber sheets (*** *p* < 0.001).

The wettability of biomaterials plays a crucial role in their biocompatibility and can significantly affect their friction properties. Typically, hydrophilic surfaces enhance lubricity [38,39]. We characterized the hydrophilicity of pristine and dual-function hydrogel coating-modified silicone rubber sheets using WCA (Figure 3). The results indicated that the silicone rubber sheets modified with dual-function hydrogel coating exhibited a much lower WCA compared to the pristine silicone sheets, which was ascribed to the incorporation of hydrophilic PVP and PEGDA. After being immersed in artificial urine for 30 days, the water contact angle of dual-function hydrogel coating-modified silicone sheets (named DF-1-S) increased to a certain extent (from 19.40 ± 0.27 to 29.22 ± 0.65), which was mainly caused by the precipitation of untangled hydrophilic polymer in the coating. Even though the contact angle increases to a certain extent, it does not affect the long-term performance of the silicone urianry catheter in combination with the antibacterial and lubricity test results.

### 2.2. Antibacterial Properties

**Figure 4:**
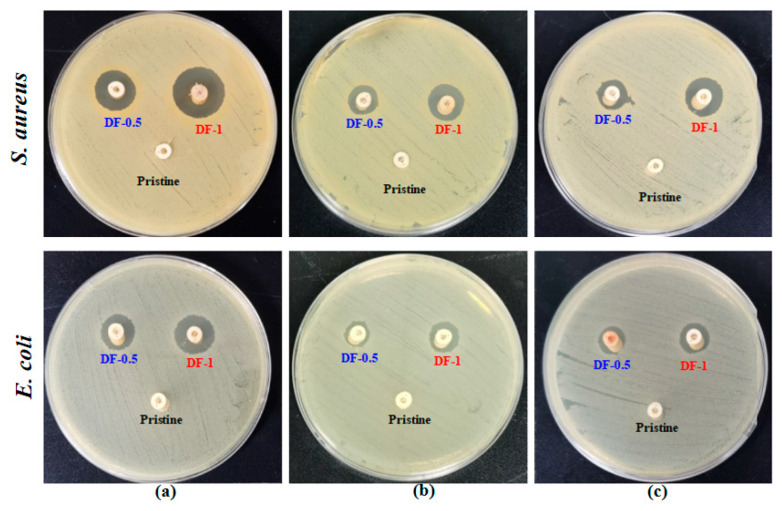
Inhibition zones of silicone urinary catheters against *S. aureus* and *E. coli* after soaking in artificial urine at various time intervals ((**a**): day 0; (**b**): day 14; (**c**): day 30).

As a typical indwelling medical device, a silicone urinary catheter is susceptible to bacterial adhesion and infection [40,41]. Hence, the antibacterial properties are very important to a silicone urinary catheter. The antibacterial properties of a pristine silicone urinary catheter and of DF-0.5 and DF-1 hydrogel coating-modified silicone urinary catheters were evaluated against *Staphylococcus aureus* (*S. aureus*) and *Escherichia coli* (*E. coli*) using the inhibition zone test, as shown in Figure 4. As anticipated, there was no inhibition zone for the pristine silicone urinary catheter, demonstrating that it exhibited no antibacterial activity against either of the bacteria, while the dual-function hydrogel coating-modified silicone urinary catheters had obvious inhibition zones at different testing times (day 0, day 14 and day 30), indicating significant antibacterial properties against *S. aureus* and *E. coli*. Additionally, it was observed that the diameters of the inhibition zones against *S. aureus* were greater than those observed against *E. coli*. This result was attributed to the structural difference in the cell wall of *E. coli* (Gram-negative bacteria) compared to *S. aureus* (Gram-positive bacteria). The Gram-negative bacteria have a highly organized cell membrane structure that can effectively resist the binding and penetration of foreign invading agents, such a chlorhexidine acetate, which induces damage to the cytoplasmic membrane and then kills the bacteria [42].

### 2.3. Lubricity Test

**Figure 5:**
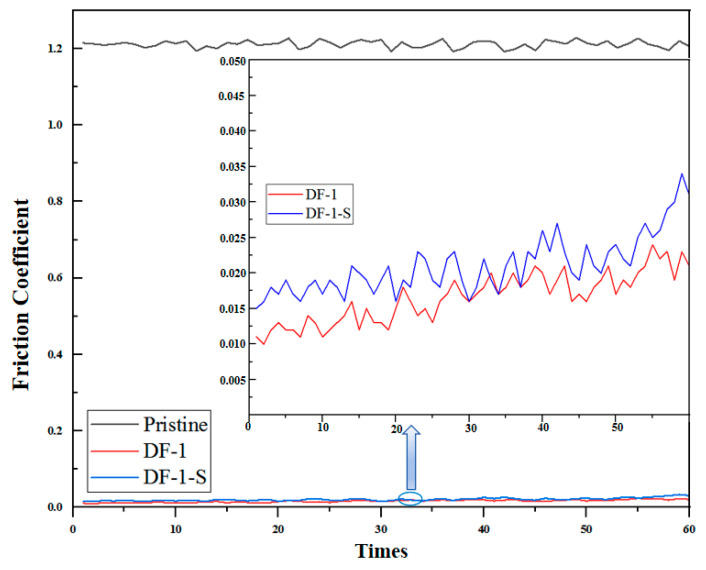
The friction coefficient test of pristine and dual-hydrogel coating-modified silicone urinary catheters.

Lubricity is a critical property for clinical application of silicone urinary catheters. The silicone urinary catheter modified with a hydrogel coating significantly minimized tissue damage, thereby improving patient safety and reducing recovery time [43,44]. We assessed the friction coefficient of both the pristine silicone urinary catheter and the dual-hydrogel coating-coated silicone urinary catheter to evaluate their lubricity, following the ISO 8295:1995 standard [45]. As illustrated in Figure 5, the friction coefficient of the dual-function hydrogel coating-coated silicone urinary catheters was dramatically reduced compared to that of the pristine silicone urinary catheters. After performing as many as 60 tests, the friction coefficient of silicone urinary catheters coated with DF-1 remained below 0.025, demonstrating the excellent adhesion and lubricity of the hydrogel coatings. To further verify the durability and lubricity of the coating, we soaked the DF-1 hydrogel coating-coated silicone urinary catheters (named DF-1-S) in artificial urine at 37 °C for 30 days to simulate their in vivo use, and then tested the friction coefficient. The coated catheters, after 60 tests, still had a friction coefficient below 0.050, indicating good adhesion and lubricity of the hydrogel coatings.

### 2.4. Cytotoxicity Assessment

**Figure 6:**
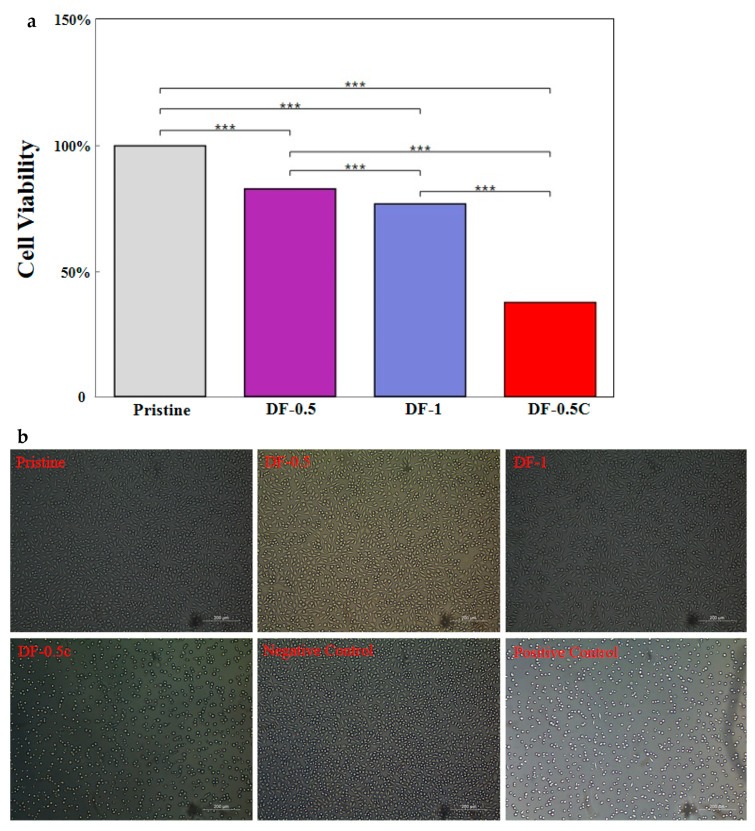
Cell viability (**a**) and morphology (**b**) of L929 cells with pristine and dual-function hydrogel coating-modified silicone urinary catheters (*** *p* < 0.001).

Using the MTT assay method and L929 cells, we examined the cytotoxicity of the pristine and dual-function hydrogel coating-modified silicone urinary catheter extracts, as outlined in ISO 10993-5:2009 [46]. As illustrated in Figure 6a, the cell viability of both the pristine silicone urinary catheters and those modified with dual-function hydrogel coatings (DF-0.5 and DF-1) remained above 70%, and the cells had normal morphology and grew well (as shown in Figure 6b), suggesting that there was no apparent cytotoxicity observed in L929 cells. Meanwhile, it can be seen from Figure 6a that the cell viability of DF-0.5C (prepared by directly dissolved chlorhexidine acetate in hydrogel coating) was below 40% and the cells were round and few in number, showing that there was severe cytotoxicity to L929 cells, which was because DF-0.5C had no PVAc carrier coating and chlorhexidine acetate was released quickly, resulting in severe cytotoxicity.

### 2.5. Vaginal Irritation Test

**Figure 7:**
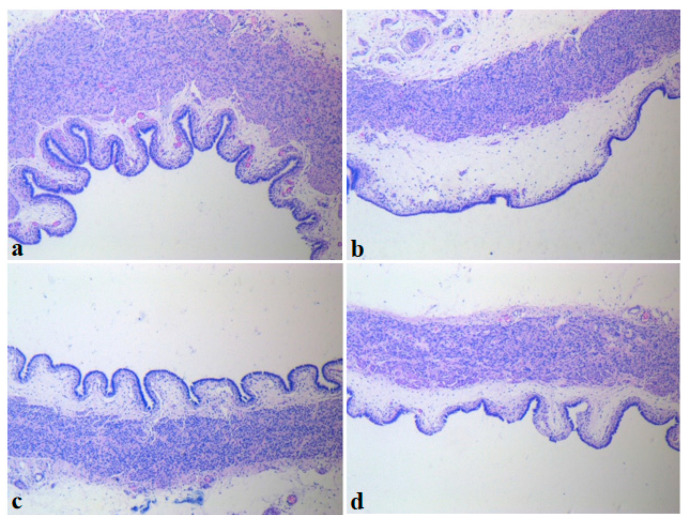
Photomicrographs of pathological sections from different groups ((**a**): polar test; (**b**): polar control; (**c**): non-polar test; (**d**): non-polar control).

The objective of this evaluation serves to assess the dual-function hydrogel coating (DF-1)-coated silicone urinary catheter’s prospect of causing irritation to vaginal tissue under the specified testing conditions, in accordance with ISO 10993-10: 2010 [47]. It is evident from Table 1 that the average scores for both the experimental and control samples were 0. Additionally, Figure 7 illustrates that the tested groups did not exhibit inflammation in the pathological section images. This indicates that the DF-1 hydrogel-coated silicone urinary catheter is unlikely to cause any potential irritation to vaginal tissue.

## 3. Conclusions

In conclusion, dual-function hydrogel coating-coated silicone urinary catheters were prepared with durable antibacterial properties and lubricity. The results from FTIR, SEM, and WCA tests demonstrated the effective modification of the silicones. The dual-function hydrogel coating markedly enhanced the silicone urinary catheters’s hydrophilicity and had enduring antibacterial activity against *S. aureus* and *E. coli*. The modified silicone urinary catheters still maintained antimicrobial activity after being soaked in artificial urine for 30 days to simulate their in vivo use. Moreover, the friction test demonstrated that the modified silicone urinary catheters had reduced friction and good durability. After being soaked in artificial urine for 30 days and subjected to as many as 60 tests, the friction coefficient remained below 0.05. Finally, the results from the cytotoxicity and irritation assessments demonstrated that the dual-function hydrogel coating-coated silicone urinary catheters exhibited superb biocompatibility, which has significant promise for application in hospitals. At the same time, the results of the inhibition zone test and cytotoxicity test confirmed the PVAc carrier coating’s sustained release of chlorhexidine acetate.

## 4. Materials and Methods

### 4.1. Materials

Chlorhexidine acetate and poly(vinyl acetate) were acquired from Shanghai Aladdin Bio-Chem Technology Co., Ltd. (Shanghai, China). Acetone and isopropanol were procured from Chengdu Kolon Chemical Co., Ltd. (Chengdu, China). Silicone urinary catheters were procured from Haiyan Kangyuan Medical Instrument Co., Ltd. (Jiaxing, China). The bacterial strains, *Staphylococcus aureus* (CMCC26003) and *Escherichia coli* (CMCC44102), were procured from Guangdong Microbial Culture Collection Center (Guangzhou, China). Tryptic soy agar was procured from Beijing SanYao Science & Technology Co., Ltd. (Beijing, China). Cottonseed oil was acquired from Shanghai Macklin Biochemical Co., Ltd. (Shanghai, China). Saline was acquired from Shandong Hualu Pharmaceutical Co., Ltd. (Liaocheng, China). MEM medium was acquired from Beijing Itta Biotechnology Co., Ltd. (Beijing, China), and 3-(4,5-Dimethylthiazol-2-yl)-2,5-diphenyltetrazolium bromide (MTT) was acquired from Tokyo Chemical Industry (Tokyo, Japan). The rest of the materials used were from the same sources as those reported in our previous literature [36].

### 4.2. Fabrication of Silicone Sheet

The PDMS Sylgard™184 Kit was blended and cured according to our previous literature [36] to prepare silicone sheets.

### 4.3. Fabrication of Dual-Hydrogel Coating-Modified Silicone Urinary Catheters and Sheets

The silicone urinary catheters and sheets were modified using a three-step dipping procedure. The primer layer and hydrogel coating layer were identical to our previous literature [36]. The silicones coated with a primer layer were immersed into the solution of 1 wt% poly(vinyl acetate), chlorhexidine acetate and acetone to form a carrier coating. For comparison, 0.05 wt% chlorhexidine acetate was directly added to the hydrogel coating without the PVAc carrier coating and named DF-0.5C.

### 4.4. Surface Characterization of Silicone Urinary Catheters and Sheets

The Nicolet iS10 FTIR spectrometer (Thermo Scientific, Waltham, MA, USA) and static WCA (Data Physics OCA, Filderstadt, Germany) were used to analyze the surface functional groups and wettability of silicone sheets, respectively. The Nova NanoSEM 450 (FEI, Hillsboro, OR, USA) was used to observe the silicone urinary catheters’ outer surface morphology.

### 4.5. Antibacterial Test

The antibacterial properties of the silicone urinary catheters were evaluated by the inhibition zone method. *S. aureus* and *E. coli* were chosen as model bacterial strains. The pristine and dual-function hydrogel-modified silicone urinary catheters were each cut into 1 cm lengths and subsequently exposed to ultraviolet (UV) irradiation for 30 min for the purpose of sterilization. Then, 30 μL of bacterial suspension (10^6^ CFU mL^−1^) was evenly spread onto the surface of tryptic soy agar (TSA), followed by placing the sterilized samples on them. The agar plates were incubated at 37 °C for 24 h, after which the zones of inhibition were measured.

### 4.6. Friction Coefficient Test

The silicone urinary catheters were cut into tubular pieces at least 20 cm in length, and the coefficient of friction was tested. Before each test, the samples were immersed in water for 1 min. After taking 2 test samples as a group, the slider was allowed to move a sufficient distance (no less than 100 mm). Then, the force-versus-displacement curve was recorded. Following each assessment, the slider was washed with fresh water to prevent any potential contamination from the coating that may have detached from the test surface.

### 4.7. Cytotoxicity Test

For the evaluation of cytotoxicity, the L929 mouse fibroblast cell (Wuhan Servicebio Biotechnology Co., Ltd., Wuhan, China) was utilized. The tested silicone urinary catheters were extracted in a complete MEM medium containing 10% FBS for some 72 h at 37 °C in an incubator with a surface area-to-volume ratio of 3 cm^2^/mL. The negative and positive control samples were extracted at concentration of 3 cm^2^/mL and 6 cm^2^/mL, respectively. The L929 cells were plated in 96-well plates and cultured for approximately 24 h to achieve a nearly confluent monolayer, after which they were subjected to the extract of the test article. After being exposed for 24 h, the extracts were discarded and the MTT solution was introduced into each well. The plate was incubated for 2 h. The MTT solution was subsequently discarded and substituted with 100 μL of isopropanol. An Infinite F50 microplate reader (Tecan, Männedorf, Switzerland) was used to assess the absorbance at 570 nm. The cell viability was determined using the equation shown below (1):

(1) $$Cellviability\left(\%\right)=\left[\frac{\left({OD}_{test}-{OD}_{nc}\right)}{\left({OD}_{pc}-{OD}_{nc}\right)}\right]\times 100\%$$

where OD_test_ represented the absorbance of the extract from the test group at a wavelength at 570 nm, OD_nc_ represented the absorbance of the negative control group at a wavelength at 570 nm, while OD_pc_ represented the positive control group’s absorbance at a wavelength of 570 nm. A cell viability of 70% or higher was regarded as non-cytotoxic.

### 4.8. Vaginal Irritation Test

The vaginal irritation of the dual-function hydrogel coating-coated silicone urinary catheters (DF-1) test samples was evaluated in New Zealand white rabbits. The samples were extracted with polar (saline) and non-polar (cottonseed oil) media with a surface area-to-volume ratio of 3 cm^2^/mL. The 1 mL extracts of the test samples were administered into the rabbits’ vaginas and maintained continuously for 5 days to monitor for any potential vaginal irritation reactions. The identical procedure was utilized for both the control group and the test subjects. Then, 24 h after the initial contact and before each test operation, the state of the vaginal orifice and perineum was observed and recorded. Following the last contact, the white rabbits were euthanized and the vaginal tissue samples were collected to assess the irritation state. The control samples were processed identically to the test samples. Each tissue sample was evaluated according to the scoring system illustrated in ISO 10993-10: 2010 [47].

The experimental animals and procedures of the vaginal irritation test were authorized by the Ethics Committee of the Tianjin customs district industrial products safety and technical center (permit number: 2300029-3). Animal welfare was closely monitored throughout the course of the experiment.

### 4.9. Statistical Analysis

All data were presented as mean ± standard deviation (SD) values. When comparing two groups, the differences were evaluated using a t-test, and *p*-values < 0.05 were deemed statistically significant (* p < 0.05, ** p < 0.01, *** p < 0.001). In this article, at least three parallel experiments were carried out for the assays.

## Figures and Tables

**Table 1:** Observation score of vaginal irritation test.

Group	Slice Position	Score				Average Score
		Epithelial	Leukocyte Infiltration	Vascular Congestion	Edema	
Polar test	Front/ Middle/ Back	0	0	0	0	0
Polar control	Front/ Middle/ Back	0	0	0	0	0
Non-polar test	Front/ Middle/ Back	0	0	0	0	0
Non-polar control	Front/ Middle/ Back	0	0	0	0	0

## Data Availability

The original contributions presented in the study are included in the article, further inquiries can be directed to the corresponding authors.
